# The multifaceted therapeutical role of low‐density lipoprotein receptor family in high‐grade glioma

**DOI:** 10.1002/1878-0261.13730

**Published:** 2024-09-14

**Authors:** Elisa Mastrantuono, Matilde Ghibaudi, Diana Matias, Giuseppe Battaglia

**Affiliations:** ^1^ Instituto de Medicina Molecular João Lobo Antunes, Faculdade de Medicina Universidade de Lisboa Portugal; ^2^ Institute for Bioengineering of Catalonia Barcelona Institute of Science and Technology Spain; ^3^ Biomedical Research Networking Center in Bioengineering, Biomaterials, and Nanomedicine (CIBER‐BBN) Madrid Spain; ^4^ Catalan Institution for Research and Advanced Studies Passeig de Lluís Companys Barcelona Spain

**Keywords:** glioblastomas, low‐density lipoprotein receptors, targeted therapies

## Abstract

The diverse roles of the low‐density lipoprotein receptor family (LDLR) have been associated with many processes critical to maintaining central nervous system (CNS) health and contributing to neurological diseases or cancer. In this review, we provide a comprehensive understanding of the LDLR's involvement in common brain tumors, specifically high‐grade gliomas, emphasizing the receptors' critical role in the pathophysiology and progression of these tumors due to LDLR's high expression. We delve into LDLR's role in regulating cellular uptake and transport through the brain barrier. Additionally, we highlight LDLR's role in activating several signaling pathways related to tumor proliferation, migration, and invasion, engaging readers with an in‐depth understanding of the molecular mechanisms at play. By synthesizing current research findings, this review underscores the significance of LDLR during tumorigenesis and explores its potential as a therapeutic target for high‐grade gliomas. The collective insights presented here contribute to a deeper appreciation of LDLR's multifaceted roles and implications for physiological and pathological states, opening new avenues for tumor treatment.

## Graphical abstract

The role of LDLR family receptors in mediating the transport of LDL across the blood–brain barrier (BBB), facilitating processes such as survival, proliferation, and invasion in high‐grade gliomas. Nanoparticles targeting LDLR can be used for drug delivery, potentially inducing cell death and reducing tumor proliferation and survival in high‐grade glioma cells.

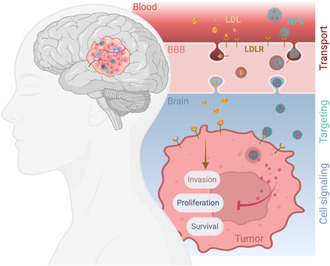

**Abbreviations**
ANG2: angiopep2
ApoE: apolipoprotein E
APOER2: apolipoprotein E receptor 2
AuNP: gold nanoparticles
BBB: blood–brain barrier
by NIR: near‐infrared
CDKN2A: cyclin‐dependent kinase inhibitor 2A
CNS: central nervous system
CSF: cerebrospinal fluid
DOX: doxorubicin
EGFR: epidermal growth factor receptor
EMT: epithelial to mesenchymal transition
GBM: glioblastoma
GNRs: responsive gold nanorods
GSCs: glioma stem‐like cells
HGA: high‐grade astrocytoma
IDH/IDH2: isocitrate dehydrogenase 1/2
JNK: c‐Jun N‐terminal kinase
LDL: low‐density lipoprotein or cholesterol
LDLR: low‐density lipoprotein receptor family
LGA: low‐grade astrocytoma
LRPs‐LDLR: related proteins
MAPK: mitogen‐activated protein kinase
NF1: neurofibromin 1
PDGFRA: platelet‐derived growth factor alpha
PEG‐PCL: poly(ethylene glycol)‐co‐poly(ε‐caprolactone)
POs: polymersomes
PTTs: photothermal therapies
PTX: paclitaxel
RMT: receptor‐mediated transcytosis
TMZ: temozolomide
WHO: World Health Organization
WNT: Wingless and Int‐1

## 1 Introduction

Among the tumors that affect the central nervous system (CNS), gliomas are the most aggressive and prevalent primary brain tumors, representing ~80% of all malignant CNS tumors. It has been hypothesized that gliomas originate from glial cells that support neurons in the brain, including astrocytes, oligodendrocytes, and ependymal cells. According to the World Health Organization (WHO), gliomas have been categorized into four grades according to their aggressiveness based on their histological characteristics. Grade III gliomas (anaplastic astrocytoma) and grade IV (glioblastoma, GBM) collectively contribute to almost half of all malignant brain tumors and are commonly called high‐grade gliomas [1, 2]. Among them, GBM is the most malignant form, with a median survival of 12 months [3]. This poor prognosis is mainly attributed to the blood–brain barrier (BBB) hindering the entry of therapeutics into the tumors’ site. Therefore, it becomes crucial to identify possible targets for drug delivery systems and allow them to efficiently cross the BBB to reach the tumors site and target the signaling pathways involved in the multiresistance of GBM. One of the possible targets is the low‐density lipoprotein receptor (LDLR) family, which have been implicit in GBM tumorigenesis. The LDL receptor family (LDLR) comprises several transmembrane receptors, including LDLR, LDLR‐related proteins‐1 (LRP1), and ‐8 (LRP8), which share a similar structure, as well as LRP5 and LRP6, which are structurally distinct members. Their function in cellular uptake and activation of signaling pathways for the proliferation and survival of tumor cells has been investigated; however, a lot still needs to be explored. LDL receptors can recognize, bind, and internalize various extracellular ligands, participating in a broad spectrum of physiological processes [4]. Numerous studies have confirmed the presence of LDL receptors in healthy and diseased brains [5, 6]. The panoply of ligands and LRPs' broad expression across the cells/tissues makes these receptors multifunctional and involved in processes from the embryonic to the adult stage. Additionally, LRPs play a role in lipid homeostasis, cell migration, proliferation, and differentiation, especially in tumorigenesis [7]. Thus, these receptors may be optimal targets for receptor‐mediated transcytosis (RMT) and glioma targeting. Although LRP expression has been identified in various cancers, its roles have not yet been entirely understood due to fluctuations in receptor‐mediated signal transduction events. This section aims to review recent advances highlighting the role of LDLRs in glioma by analyzing the involvement of select LRPs in pathways leading to tumorigenesis and their potential role in targeted therapies.

## 2 LDLRs as key players for glioma progression

LDLRs are key regulators of various physiological processes in both healthy and disease states. Among all the receptors in this family, LDLR and LRP1 are well‐known to be overexpressed in GBM tissues as compared to normal brain tissues [8, 9], suggesting their potential key role in regulating tumor aggressiveness and malignant potential, and thus in tumor progression. In this section we will explore how these processes are regulated through the cellular uptake and transport of molecules, as well as the signaling pathways mediated by LDLRs in glioma.

### 2.1 Cellular uptake and transport of molecules

The most investigated function associated with LDL receptors is the regulation of cholesterol homeostasis and metabolism through the endocytosis of LDL cholesterol [6]. LDL molecules bind to these receptors on the extracellular membrane, and their internalization occurs via the clathrin‐dependent pathway, initiating lipid metabolism inside the cells [10]. In this context, cancer cells exhibit an aberrant regulation of cholesterol homeostasis, with an enhanced LDL uptake. This deregulation contributes to several aspects of tumor cell growth and energy production. Cholesterol is also required for new membrane synthesis, being crucial in the case of highly proliferative cells such as neoplastic cells [11]. The cerebrospinal fluid (CSF) lipid profile in grade III glioma revealed a substantial elevation in the levels of 1‐oleyl cholesterol and tetrahydrocorticosterone, suggesting a potential connection to malignant transformation. Additionally, the serum of GBM patients exhibits high levels of LDL, a factor associated with the growth and proliferation of tumors [12]. Indeed, due to their faster cell growth and proliferation, GBM cells have higher cholesterol needs, which are fundamental for membrane synthesis, energy production, and other cellular processes. For this reason, LDL uptake and metabolism are usually dysregulated in tumors to support their growth and proliferation, and receptors involved in LDL uptake exhibit dysfunctions or mutations [13]. In the brain, the BBB poses a challenge to the penetration of LDL molecules, which are primarily internalized through LDL receptors or generated via intracellular biosynthesis. Notably, a downregulation of enzymes related to cholesterol synthesis has been observed in glioma cells [12]. This, together with the presence of the BBB, implies that the primary source of cholesterol for glioma cells is receptor‐mediated transcytosis (RMT), such as LDLRs. Hence, LDLR activity is crucial for glioma cell survival. Different GBM cell lines (including SF‐767, SF‐763, A‐172, U‐87 MG, U‐251 MG, U‐343 MG, and SF‐539) have been studied, demonstrating a high expression of LDLRs, specifically of LDLR and LRP1 receptors, which could then be the main importers of cholesterol [14]. The overexpression of LDLR and LRP1 can lead to higher intake of LDL in the cells, providing more energy to sustain the uncontrolled growth of tumor cells.

#### 2.1.1 LDLR

**Fig. 1:**
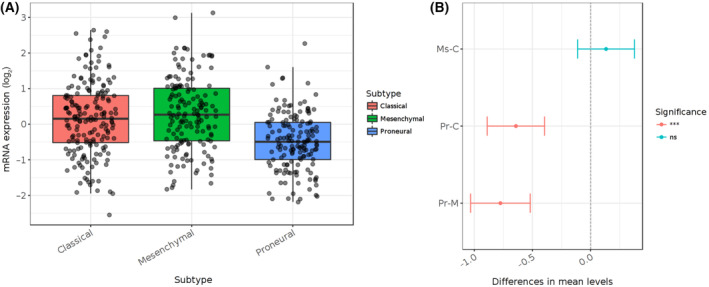
LDLR expression in subtypes of GBM. (A) The LDLR mRNA levels were analyzed and compared among subtypes of GBM samples (classical *N* = 182, mesenchymal *N* = 156 and proneural) samples, based on TCGA_GBM dataset. (B) The diagram on the right illustrates variances among pairs, featuring the 95% confidence interval and the *P*‐values associated with the pairwise comparisons. ***P < 0.001 by Tukey's Honest Significant Difference test [18]. GBM, glioblastoma; LDLR, low‐density lipoprotein receptor family; TCGA, The Cancer Genome Atlas Program.

Recently, based on transcriptional signatures, GBMs have been categorized into Classical, Proneural, and Mesenchymal subtypes. A favorable outcome is more frequent in the Proneural and Classical subtype, while poor prognosis and survival is associated with the Mesenchymal subtype [15]. Several mutations have been identified in these subtypes, reflecting the heterogeneity of GBMs. In the Proneural subtype, mutations in isocitrate dehydrogenase 1 and 2 (IDH/IDH2) and tumor suppressor 53 (TP53), along with platelet‐derived growth factor alpha (PDGFRA) amplification, are frequently observed. The Mesenchymal subtype presents mutations in neurofibromin 1 (NF1) and TP53, as well as CDKN2A deletions. The Classical subtype is characterized by PTEN mutations, which are responsible for PI3K/AKT pathway activation, amplification of epidermal growth factor receptor (EGFR), and deletion of cyclin‐dependent kinase inhibitor 2A (CDKN2A) [16]. For instance, EGFRvIII, a common EGFR mutation found in the Mesenchymal subtype―the most aggressive form of GBM―has been shown to upregulate LDLR expression [17]. Data from The Cancer Genome Atlas (TCGA) further indicate that LDLR expression is more prevalent in both the Mesenchymal and Classical subtypes of GBM, highlighting its potential role in tumor aggressiveness and overall survival (Fig. 1).

This increased expression is associated with a higher proliferation rate and enhanced survival of tumor cells due to the increased levels of internalized LDL [19]. Additionally, research has demonstrated a correlation between LDLR expression and the malignancy grade in GBM cells, with higher expression levels linked to greater tumor severity [9]. These results suggest that LDLR may play a pivotal role in tumor aggressiveness and overall survival, with more aggressive subtypes showing higher receptor expression. Therefore, targeting LDL uptake mechanisms could offer potential treatment for GBMs.

#### 2.1.2 LRP1

**Fig. 2:**
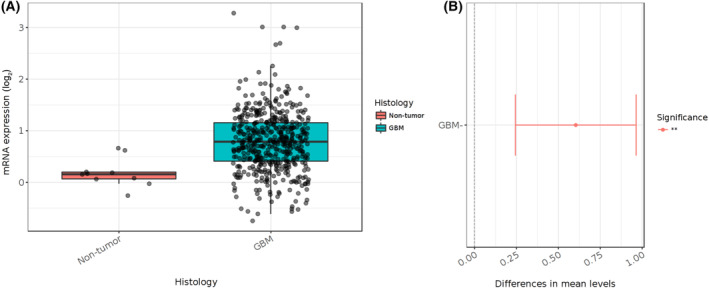
mRNA levels of LRP1 in GBM. (A) The LRP1 mRNA was analyzed in 486 GBM samples and compared to nontumor samples (*n* = 10), according to TCGA. The mRNA levels of LRP1 were a 1.06‐fold‐change in GBM samples as compared to nontumor. (B) The diagram on the right illustrates variances among pairs, featuring the 95% confidence interval and the *P*‐values associated with the pairwise comparisons. **P < 0.01 by Tukey's Honest Significant Difference test [18]. LRP1, LDLR‐related protein 1.

LRP1 has been associated with the poor prognosis of GBM. For instance, LRP1 mRNA levels are higher in GBM compared to nontumor samples, as indicated by the TCGA data (Fig. 2). Several studies have investigated the role of LRP1 in the survival and prognosis of GBM [4]. Shruthi and collaborators explored the correlation between LRP1 expression, glioma cells' cholesterol content, and the tumor's aggressiveness. Notably, GBM cases exhibit significantly LRP1 higher expression than low‐grade astrocytoma (LGA) and high‐grade astrocytoma (HGA) cases, suggesting a correlation between LRP1 expression and the poor outcome of the tumors. Furthermore, it was demonstrated that the inactivation of the LRP1 receptor leads to a significant reduction in the total cholesterol content in the cells and a decrease in cell viability, proliferation, and invasiveness in an *in vitro* GBM model [4, 9].

The identified upregulation of LDLR and LRP1 receptor expression establishes a link to the intracellular cholesterol uptake in tumor cells, contributing to the increased proliferation rate observed in these cells. The subsequent section focuses on the intricate role of some LRPs in signaling pathways associated with tumors cell growth, specifically addressing the case of glioma cells.

### 2.2 Signaling pathways mediated by LRPs in gliomas

#### 2.2.1 LRP1 and LRP8 in mitogen‐activated protein kinase (MAPK) pathways

Mutations and dysregulation of MAPK pathways, particularly ERK1/2, p38 MAPK and c‐Jun N‐terminal kinase (JNK) are observed in different subtypes of GBM, and correlated with cell differentiation, invasiveness, and metastatic behavior [20]. The aggressive behavior of the Mesenchymal subtype is due to the high levels of p38MAPK, ERK, and JNK signaling [21]. Some studies have highlighted the role of specific LDLRs in regulating these pathways, suggesting a potential role in tumorigenesis. In GBM, silencing of LRP1 was associated with a decreased level of phosphorylated ERK‐1/2 and increased levels of phosphorylated JNK‐1/2/3 [22], suggesting a potential pivotal role of LRP1 in regulating intracellular signal transduction involved in cancer progression. Specifically, upregulation of the ERK pathway in GBM via the LRP1 receptor has been associated with increased production of various metalloproteinases (MMPs), which facilitate cell migration and invasion [23]. Conversely, inhibition of the JNK pathway is associated with reduced apoptotic processes, allowing cancer cells to proliferate [24]. Consequently, silencing of LRP1 has been linked to decreased MMP‐2 and MMP‐9 levels, which inhibits GBM cell proliferation. In addition to LRP1, the LRP8 receptor, also known as Apolipoprotein E Receptor 2 (APOER2), has been implicated in the regulation of MAPK pathways. Widely expressed in the CNS, LRP8 plays a vital role in several physiological processes, including neuronal development [25]. Numerous studies have highlighted LRP8's involvement in tumorigenesis across different cancers, with its overexpression often correlating with poor patient prognosis [26, 27, 28]. Gene ontology analysis based on breast cancer cell lines has underscored the significant impact of LRP8 knockdown on the MAPK pathways. Due to its influence on these pathways, LRP8 silencing inhibited epithelial to mesenchymal transition (EMT) and sensitized cancer cells to chemotherapy [28]. Since GBM tumors also display EMT‐like behavior, further investigation is needed to understand the intricate roles of LRP8 during this process. Evidence of LRP8 upregulation in breast stem‐like cells suggest its potential involvement in the stemness and invasiveness of GBM cells, as well [28].

#### 2.2.2 LRP5 and LRP6 in the Wnt/β‐catenin signaling pathway

Other LDLRs involved in cellular processes are LRP5 and LRP6, coreceptors of the Wingless and Int‐1 (Wnt)/β‐catenin signaling pathway, which plays a vital role in cellular proliferation, migration, and invasion [29]. Upon the activation of Wnt signaling, phosphorylated LRP5/6 facilitates the translocation of β‐catenin into the nucleus, activating the expression of genes and transcription factors that promote EMT. Cells undergoing EMT acquire stem cell properties, leading to metastatic behavior and higher therapy resistance [8, 30, 31]. These processes are primarily known for playing an essential role in the progression of epithelial tumors. However, more recently, EMT‐like processes have also been investigated in nonepithelial tumors, such as gliomas, hematopoietic malignancies, and sarcomas [32]. Certain studies have reported overexpression of Wnt proteins in human malignant glioma, establishing a link between this upregulation and the proliferation of glioma cells [33]. Furthermore, it has been shown that GBM exhibits higher LRP6 expression than normal brain tissues [34]. These findings underscore the role played by LRP5/6 coreceptors in promoting the Wnt/β‐catenin signaling pathway, leading to an EMT‐like process and brain cancer progression. Hence, LRP5 and LRP6 emerge as promising therapeutic targets. Indeed, it was demonstrated that blocking the interaction between Wnt proteins and LRP5/6 coreceptors lead to significant antitumor effects [35]. Additionally, exploring the modulatory function of microRNAs on the Wnt/β‐catenin signaling pathway, a recent study demonstrated that miR‐137 targets LRP6 and effectively inhibits GBM growth and temozolomide (TMZ) resistance [34]. Besides, LRP8 also positively regulates the Wnt/β‐catenin signaling pathway by promoting the stabilization and nuclear accumulation of β‐catenin [7]. Indeed, triple depletion of LRP8 and LRP5/6 might promote a more potent inhibition of the Wnt signaling, compared with only LRP5/6 knockdown [36].

Moreover, the Wnt signaling pathway also plays a crucial role in developing vasculature in the CNS and maintaining the integrity of the BBB [37]. The activation of the Wnt pathway through LRP5/6 contributes to the formation of tight junctions, and its knockdown can result in the disorganization of regular cell–cell junctions [38]. Indeed, it has been observed that even a modest reduction in Wnt signaling in brain endothelial cells could impact the maintenance of the BBB and the development of its vasculature [39]. A recent study noted increased TMZ delivery and enhanced glioma sensitivity to chemotherapy after inhibiting the Wnt pathway [40], possibly due to the loss of BBB integrity. Therefore, discovering ways to modulate BBB permeability could significantly influence drug delivery systems for brain tumors, enhancing the overall effectiveness of treatment. Reducing LRP expression in brain endothelial cells might be a plausible approach to achieve this. Nevertheless, the correlation between BBB permeability, Wnt signaling, and LRP receptors is not fully understood, and its potential implications for cancer treatment are still under research.

After delving into the intricate involvement of LDLRs in various biological processes correlated with tumorigenesis, assessing how they can be exploited in targeted therapies is essential.

## 3 LRPs in targeted therapies for glioma

**Fig. 3:**
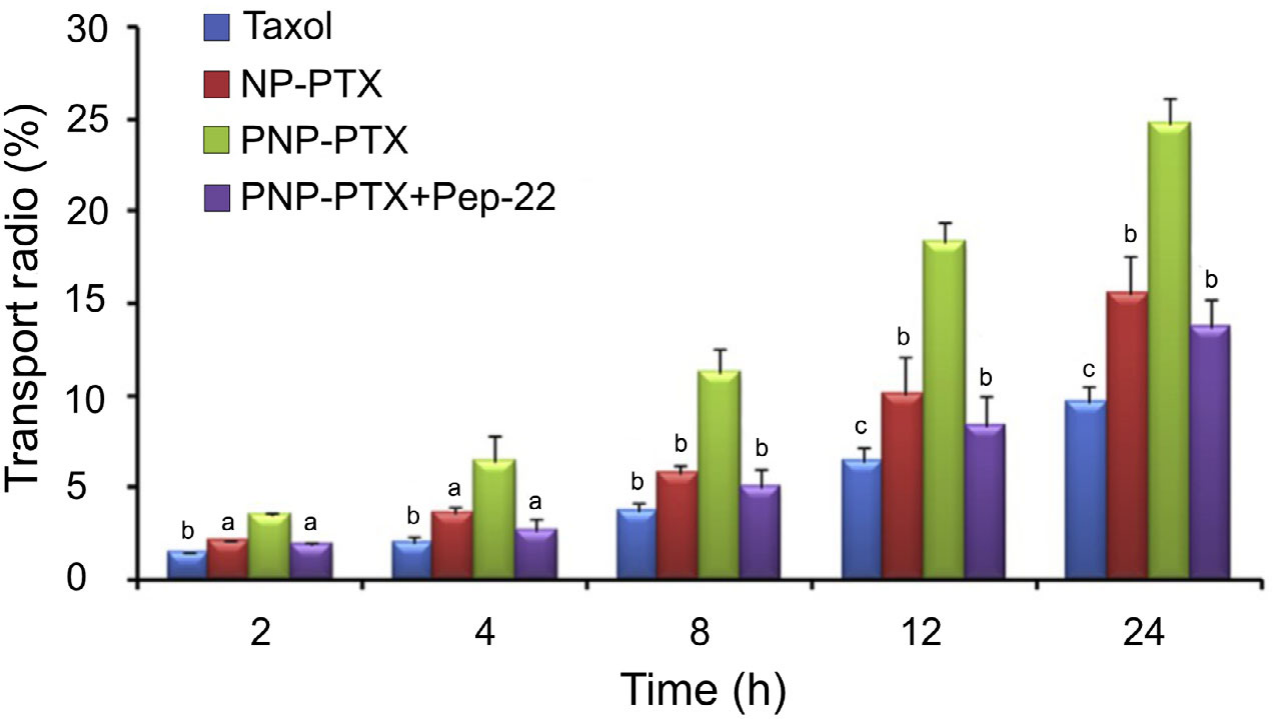
Transport ratio of different formulation across an *in vitro* model of the BBB during 24 h. NP‐paclitaxel (PTX): NPs without the functionalization, PNP‐PTX: NPs functionalized with peptide‐22, PNP‐PTX + Peptide‐22: NPs functionalized + pre‐incubation with excess peptide‐22. Data are shown as mean ± standard deviation (*n* = 3). ^a^ *P* < 0.05, ^b^ *P* < 0.01, ^c^ *P* < 0.001, *vs* PNP‐PTX by Student's *t*‐test. Figure reproduced from Ref. [46]. Copyright © 2013 Elsevier Ltd. NP, nanoparticlesl PTX, paclitaxel.

The multifunctionality of LDLRs in triggering various cellular responses and their crucial role in the progression of different cancers make them valuable targets for therapeutic approaches. Among these receptors, LDLR and LRP1 have been extensively studied in the context of targeted therapies for glioma. This is due to their well‐documented overexpression in glioma, but also in endothelial cells of brain capillaries, compared to normal brain parenchyma [8, 41, 42, 43]. Given the high selectivity of the BBB for major cancer drugs, researchers have been developing new therapeutic strategies that leverage the high expression of LDLR and LRP1 in the BBB. Recent studies have focused on LDLR as a potential target for GBM using various types of nanoparticles. A specialized drug delivery system using polymeric micelles, which present the apolipoprotein E (ApoE) peptide to target LDLR, was developed to encapsulate sorafenib, a kinase inhibitor that blocks the RAF–MEK–ERK (MAPK) pathway and inhibits cancer cell proliferation. This system significantly increased both drug uptake and its cytotoxic effects compared to free sorafenib [44]. A similar outcome was achieved by using gold nanoparticles (AuNP) to enhance the effectiveness of proton therapy. A significantly higher anticancer impact of LDLR‐targeting AuNP than bare AuNP was observed [45]. As shown in Fig. 3, the involvement of LDLR in BBB penetration was further assessed through the saturation of binding sites using a specific ligand, peptide‐22. The transport ratio of functionalized NPs decreased after preincubation with peptide‐22, underscoring the pivotal role of LDLR in mediating the transport of NPs across the BBB [46]. These findings suggest a potentially significant role of this receptor in targeted therapies for brain diseases, particularly in glioma treatment.

The LRP1 receptor has gained much more attention in cancer research in the last years due to its great expression in glioma cells and to its capacity to induce receptor‐mediated transcytosis. Notably, LRP1 exhibits higher overexpression compared to other LDLRs, as documented in previous studies [4], suggesting the potential for more specific targeting. Among the variety of ligands binding to LRP1 receptor, angiopep2 (ANG2) peptide has emerged as a focal point in research over the years, with several studies demonstrating its ability to cross the BBB and effectively target GBM cells [47, 48, 49]. The interaction between ANG2 and the LRP1 receptor has been harnessed to enhance the uptake of various types of carriers, improving the overall efficacy of different therapies. Nanoparticle‐based therapies have emerged as a promising approach to improve GBM treatment, exploiting the LRP1 receptor as a target to cross the BBB and deliver drugs to GBM cells. In Table 1, we summarize the studies of the last 5 years that have investigated nanoparticle‐based therapies for GBM. The studies highlight the potential of nanotechnology to overcome the limitations of traditional chemotherapy and radiotherapy, offering hope for improved patient outcomes.

**Table 1:** The most recent therapeutical approaches to target LRP1 receptor with ANG2. ANG, angiopep2; BBB, blood–brain barrier; CS, chitosan; DOX, doxorubicin; EP‐1, EGFR‐targeting peptide‐1; EXO, exosome; GBM, glioblastoma; LP‐MSN, lipid‐coated mesoporous silica nanoparticle; MNP, magnetic nanoparticles; MRI, magnetic resonance imaging; NIR, near‐infrared; PCL‐PEOz, polycaprolactone‐poly‐(2‐ethyl‐2‐oxazoline); PTA, photothermal activator; PTT, photothermal therapy; PTX, paclitaxel; SA, stearic acid; SAP, saporin; SDT, sonodynamic therapy; TAT, transactivator of transcription peptide; TMZ, temozolomide.

Reference	Nanoparticle	Treatment	LRP1 role
[50]	Micelles	Cytotoxicity induced by DOX loaded on ANG micelles together with radiosensitizer Dbait	BBB crossing and GBM targeting
[51]	Liposomes	Synergistic effect of autophagic inhibition and SDT with nanosensitizer (ANG‐modified liposomes)	BBB crossing and GBM targeting
[52]	Polymeric nanoparticles (POs)	Cytotoxicity induced by DOX loaded on ANG nanoparticles	BBB crossing and brain metastasis targeting
[53]	Magnetic nanoparticles conjugated to engineered exosomes	Ferroptosis enhancement by using MNP conjugated to ANG‐EXO	BBB crossing and GBM targeting
[54]	Gold nanorods	PTT exploiting NIR‐responsive gold nanorods as PTA	BBB crossing and GBM targeting
[55]	Liposomes	Cytotoxicity induced by DOX loaded in TAT‐ANG‐Liposomes	Targeted with ANG to cross the BBB and target GBM cells. They also used TAT peptide to overcome the limitation of receptor saturation.
[56]	Lipid coated mesoporous silica nanoparticles	Cytotoxicity induced by PTX loaded in ANG‐LP‐MSN nanoparticles	BBB crossing and glioma targeting
[57]	pH‐sensitive PCL‐PEOz polymersomes (POs)	Gold nanoparticles and DOX as a complex drug, encapsulated in ANG‐POs. Gold NPs: work as radiosensitizer to enhance the effect of radiotherapy, DOX has a cytotoxic effect on cancer cells	BBB crossing and GBM targeting
[58]	Polymersomes	Cytotoxicity induced by DOX encapsulated in ANG‐PEG‐PCL	Targeted with ANG to cross the BBB and target GBM cells
[59]	Polymeric micelles	Gene delivery to improve radiotherapy	Targeted with ANG to cross the BBB and target glioma cells
[60]	Lipid‐poly nanoparticles	Cytotoxicity induced by TMZ encapsulated in A2‐P(MIs)25 NPs, combined with the enhanced RT sensitivity caused by Mis (hypoxic cell radiosensitizers)	Targeted with ANG to cross the BBB and target glioma cells
[61]	Dendrimer‐based drug carrier	Cytotoxicity induced by DOX encapsulated in PAMAM dendrimer‐based NPs functionalized with ANG and an EP‐1	Targeted with ANG to cross the BBB
[62]	Ferritin nanoparticles	Cytotoxicity induced by DOX encapsulated in ferritin‐HREV107^5^‐Angiopep‐2 (Fn‐Rev‐Ang) nanoparticles. Imaging system by loading MRI contrast agent GdDTPA into the Fn‐Rev‐Ang NPs	Targeted with ANG to cross the BBB and target low‐grade glioma cells
[63]	Polymersomes	Cytotoxicity induced by SAP loaded on ANG‐POs	Targeted with ANG to cross the BBB and target low‐grade glioma cells
[64]	Copolymer micelles	Cytotoxicity induced by DOX loaded on ANG‐CS‐SA copolymer micelles	Targeted with ANG to cross the BBB and target low‐grade glioma cells

To overcome the poor delivery of doxorubicin (DOX) in the brain, poly(ethylene glycol)‐co‐poly(ε‐caprolactone) (PEG‐PCL) polymersomes (POs) decorated with ANG2 and loaded with DOX were used to cross the BBB and target GBM cells. ANG2‐decorated POs showed stronger accumulation and enhanced tumors cell uptake compared to the nontargeted POs. Furthermore, ANG‐POs‐DOX significantly increased the overall survival of glioma‐bearing rats [58]. LRP1 targeting has also been employed to improve the efficacy of photothermal therapies (PTTs), by conjugating ANG2 peptide to near‐infrared (NIR)‐responsive gold nanorods (GNRs). In this case as well, a higher binding of the LRP1‐targeting nanorods was observed, allowing a more efficient treatment [54]. Another study showed the effect of DOX loaded on ANG‐polymeric NPs also on brain metastatic cells, leading to an enhanced survival of mice bearing brain metastases [52].

Currently, some ANG‐drug conjugates are already in clinical trials for brain cancers, demonstrating the potential of the ANG‐LRP1 interaction in brain targeted therapies (Table 2). Besides the ANG peptide, other ligands, such as RAP12, RAP22, and lactoferrin (LF), have been used to trigger RMT and target glioma cells (Table 3). Micelles decoration with RAP12 has been exploited to deliver paclitaxel (PTX) to glioma cells, inducing much more intensive tumor apoptosis *in vivo* when compared to free PTX [65]. Moreover, LF was demonstrated to efficiently target the LRP1 receptor, leading to the internalization of the carrier and an enhanced effect of the therapy [66].

**Table 2:** Therapeutic approaches to target LRP1 receptor with ANG in clinical trial (https://clinicaltrials.gov/)

Condition	Treatment	Phase
Recurrent high‐grade glioma	ANG1005 (three paclitaxel molecules covalently linked to AND)	Phase II (NCT01967810)
Brain metastases from non‐small cell lung cancer (NSCLC)	GRN1005 (ANG conjugated with PTX)	Phase II (NCT01497665)
Brain metastases from breast cancer	GRN1005	Phase II (NCT01480583)
Brain metastases from breast cancer	ANG1005	Phase II (NCT02048059)

**Table 3:** Therapeutic approaches to target LRP1 receptor with other ligands (last 5 years)

Nanoparticle	Treatment	LRP1 role	Reference
Polymeric micelles (PEG–PLA)	Cytotoxicity induced by PTX loaded in RAP12‐PEG–PLA and RAP22‐PEG–PLA micelles	Targeted with RAP22 and RAP12 peptides to cross the BBB and target GBM cells	[65]
LF‐DHA‐ICG/NPs	Ferroptosis induced by a synergistic effect of DHA and the photosensitizer ICG, which improves phototherapy	Targeted with LF to cross the BBB and target GBM cells	[66]

## 4 Conclusions and perspectives

The LDLR family plays a crucial role in the progression of gliomas, influencing various mechanisms relevant to tumor progression, such as proliferation, migration, and survival. Current research focuses on targeting LDLRs to facilitate drug delivery across biological barriers (such as the BBB) or to directly impact glioma cells.

One way to benefit from the expression of LDLRs in tumor cells is by using nanoparticles. Nanoparticles can be employed to treat gliomas due to their effectiveness as drug delivery systems or their ability to cross the BBB. Specifically, polymeric nanoparticles have been used in recent research because of their easily customizable design characteristics, such as chemistry and functionalization using proteins, antibodies, or peptides on their surface. Overall, the comprehensive exploration of LDLR and LRP1 receptors in the context of targeted therapies suggests promising avenues for new treatment strategies in glioma or even in other neurological pathologies (i.e. Alzheimer's, Parkinson's, or stroke). These findings underscore the potential for improved specificity and increased therapeutic efficacy by targeting these receptors. Although other members of the LDLR family have demonstrated great relevance and crucial roles in resistance mechanisms, the functions of LRP5/6 or LRP8 have yet to be widely explored in gliomas. Consequently, research efforts directed at unraveling the roles of LRP5/6 or LRP8 and pave the way for designing novel therapeutic interventions for gliomas are essential. We hypothesize that their expression may also differ among GBM subtypes or even at different stages of the disease. Thus, clarifying their roles could be helpful for personalized medicine. Furthermore, during the development of tumors, there is significant interaction with stroma cells, such as immune cells, where LDLRs may play a role. Thus, how does LDLR influence the activation of immunosuppressive behavior of these tumors? Can nanoparticles targeting LDLRs be valuable tools to manipulate the infiltrative immune cells? Further research is needed to elucidate these mechanisms and determine the potential of LDLR‐targeting nanoparticles in modulating the immune response within the tumor microenvironment, which could lead to innovative therapies for gliomas.

## Conflict of interest

The authors declare no conflict of interest.

## Author contributions

EM, MG, and DM wrote the original version of the article. DM, MG, and GB designed and revised the final version. All authors have read and agreed to the published version of the article.
